# Evaluation of the Sex-and-Age-Specific Effects of PM_2.5_ on Hospital Readmission in the Presence of the Competing Risk of Mortality in the Medicare Population of Utah 1999–2009

**DOI:** 10.3390/jcm8122114

**Published:** 2019-12-02

**Authors:** Claire L Leiser, Ken R Smith, James A VanDerslice, Jason P Glotzbach, Timothy W Farrell, Heidi A Hanson

**Affiliations:** 1 Population Sciences, University of Utah, Huntsman Cancer Institute 2000 Circle of Hope Dr, Ste. 1500, Salt Lake City, UT 84112, USA; ken.smith@fcs.utah.edu (K.R.S.); heidi.hanson@hci.utah.edu (H.A.H.); 2 Department of Epidemiology, University of Washington, Seattle, WA 98105, USA; 3 Department of Family and Consumer Studies, University of Utah, Salt Lake City, UT 84112, USA; 4 Department of Family and Preventive Medicine, University of Utah, Salt Lake City, UT 84112, USA; jim.vanderslice@utah.edu; 5 Department of Surgery, University of Utah, Salt Lake City, UT 84112, USA; jason.glotzbach@hsc.utah.edu; 6 Department of Medicine, University of Utah, Salt Lake City, UT 84112, USA; timothy.farrell@hsc.utah.edu; 7 Veteran’s Affairs Salt Lake City Geriatric Research, Education, and Clinical Center, Salt Lake City, UT 84112, USA

**Keywords:** particulate matter, air pollution, readmission, mortality

## Abstract

Acute ambient air pollution exposure increases risk of cardiac events. We evaluated sex-and-age-specific effects of PM_2.5_ on hospital readmission and death among 19,602 Medicare beneficiaries (*N_events_* = 30,510) who survived cardiovascular events including myocardial infarction (MI), heart failure (HF), ischemic heart disease (IHD), and cardiac arrhythmias in Utah from 1999–2009. Fine and Gray regression jointly modeled the effect of PM_2.5_ on readmission hazard rates while allowing for the competing risk of death. Models were stratified by age and sex and adjusted for Charlson Comorbidity Index, dual enrollment in Medicare Parts A and B, temperature, and household income. There were 2032 cardiac readmissions and 1420 deaths after discharge. Among males age 65–74 years admitted for HF, a 10 μm/m^3^ increase in PM_2.5_ was associated with a 30% increase in risk of readmission (*p* = 0.01). Among females age 75–84 admitted for HF, PM_2.5_ was associated with a 22% increase in risk of readmission (*p* = 0.01). Among females age 75–84 years admitted for IHD, PM_2.5_ was associated with a 25% decrease in readmission (*p* = 0.01), however this was explained by a 36% increase in risk of death (*p* = 0.01). Exposure to PM_2.5_ was associated with increased risk of readmission and death. Associations were dependent upon age, sex, and index condition.

## 1. Introduction

A large body of evidence shows that ambient air pollution contributes to cardiovascular morbidity and mortality [1,2,3,4]. Numerous studies have shown positive associations between exposure to fine particulate matter (PM_2.5_ < 2.5 μm in aerodynamic diameter) and cardiovascular hospital admissions [3,5,6]. Only a small number of studies have focused on hospital readmissions, a national priority area to improve patients’ transitions across the continuum of care, avoid hazards of admission (particularly for older adults), and reduce costs to Medicare associated with unnecessary admissions. There is also a lack of research investigating differences in susceptibility across subpopulations, information of which could be used to target subgroups at highest risk readmission during episodes of high levels of air pollution. Individuals over the age of 65 are often treated as a homogenous population, when in fact there are known differences in risk of cardiovascular events by age and sex. The objective of this study was to evaluate the sex-and-age-specific effects of PM_2.5_ exposure on subsequent 30-day readmission and mortality in the Medicare population of cardiovascular patients in Utah.

The burden of adverse health effects due to ambient PM_2.5_ is not shared equally within populations. Older adults (age 65 years and older) and females may represent two subgroups that are particularly vulnerable to the effects of PM_2.5_. Compared to younger adults, older adults exhibit homeostenosis, where decreased physiological, metabolic, and compensatory reserves make them more susceptible to insults including air pollution [7,8]. The response to air pollution in older adults may be the result of a number of factors including early mitochondrial damage related to pollution related nanoparticle exposure, smoking history, occupational exposures, past environmental exposures, previous infections, age-related declines in immune response, impaired homeostasis, higher deposition of PM in diseased airways, antioxidant and nutritional status, comorbidities, and medication [8,9]. These factors, in combination with elevated risk for cardiovascular events with age, result in more sensitivity to PM_2.5_ exposure in older adults in relation to younger adults [10,11,12]. Sex may also be an important effect modifier and some studies have found stronger health effects of air pollution in females [13,14]. Sex differences in biological factors (e.g., lung size, particle deposition) as well as sociological gender norms (e.g., occupation, activity patterns) may play key roles [14,15]. These factors suggest that health outcomes among older individuals such as cardiovascular conditions and the associated utilization of the health system for these conditions may be significantly impacted by poor air quality, and more epidemiological research is needed to better understand the health effects of air pollution in this vulnerable population [12].

Hospital readmission rates among Medicare beneficiaries for high readmission-risk diagnoses range from 16.9% (pneumonia) to 22.0% (congestive heart failure) and are particularly costly [16,17]. Approximately 17.3% of Medicare discharges result in readmission within 30 days and account for nearly $15 billion in spending [18,19]. As a result, hospitals with higher than expected rates of readmission face financial penalty [20] and identifying risk factors for readmission is necessary to target cost reduction and improve quality of care. Although there is a considerable literature on the adverse health effects related to PM_2.5_ exposure [21], research is limited on the short-term effects of exposure on the survivors of cardiac events, who may be at risk for recurrent events when exposed to PM_2.5_. Results from previous studies suggest that individuals with a prior cardiac event are likely to have subsequent events when exposed to PM_2.5_ [22,23,24,25,26], possibly due to higher baseline risks. Exposure to PM_2.5_ may thus represent a modifiable risk factor for readmission rates. In studies of older adults, the risk of death is high due to increased age and comorbidities and failure to consider death as a competing risk can lead to biased results [27]. Because PM_2.5_ is associated with mortality in this population [2], the competing risk of mortality is an important consideration that previous studies have not addressed.

This study takes advantage of wide variation in air quality resulting from the unique topographic features of the study area. Utah’s Wasatch Front is an urban area of valleys surrounded by mountainous terrain where temperature inversions lead to acute variations in levels of ambient PM_2.5_. This setting allows us to investigate the role of acute exposure to PM_2.5_ on 30-day hospital readmission among Medicare beneficiaries. It is possible that high levels of pollution are also associated with excess mortality risks. Failing to jointly consider mortality may lead to biased results because in the most extreme cases, no readmission will be observed. We are the first study to investigate the relationship between air quality and readmissions while considering the competing risk of death. We hypothesize that the magnitude of the association will increase with age and that we will observe sex differences in the risk for readmission, with females being more sensitive to exposure to high levels of PM_2.5_.

## 2. Materials and Methods

### 2.1. Data

Cardiovascular-related admission data was obtained from Centers for Medicare and Medicaid Services (CMS). Patients age 65 and older were selected for inclusion in the study. We identified primary diagnoses of any cardiac condition from 1999–2009 and the most common cardiac hospitalizations in our data were myocardial infarction (MI), ischemic heart disease (IHD), heart failure (HF), and cardiac arrhythmias. Included ICD codes are displayed in Table S1. Individuals were included in the study if they resided in the Wasatch Front, a collection of four contiguous urban counties where the majority of Utah’s population resides. Patients were required to be enrolled in Medicare for one year prior to their admission date. Patients were required to be alive at the time of discharge and discharged to home/self-care, home care with an organized home health service organization, home under care of a home IV drug therapy provider, or home hospice. Patients with a planned readmission were treated as not readmitted [28]. Duration was measured as days from discharge to 30 days post-discharge.

### 2.2. Air Pollution Measures

We obtained 24-h average PM_2.5_ data from the United States Environmental Protection Agency’s (EPA) Air Quality System (AQS) Data Mart [29]. The study region was divided into air basins, a region likely to share a similar composition of air pollution, based on topography. Average daily PM_2.5_ concentrations were estimated for the population centroids (the point within each zip code where the population is centered) of each of the 123 zip codes in the region using inverse distance weighting of the data from all reporting monitoring stations located within or proximate to the air basin containing that centroid [30]. Inverse distance weighting is a spatial interpolation method that averages values from the monitoring stations and assigns higher weight to areas that are closer in proximity to the point of pollution measurement. Because the period of highest risk for hospital readmission is generally within the first 7 to 14 days after discharge and given previous inconsistent findings regarding how health outcomes vary by the exposure time window in an older adult population [30], we tested for differences in readmission risks by four acute exposure time intervals: Lag0 (day of), Lag1 (day before), 3-day average (average of day of and two preceding days), and 7-day average (average of day of and six preceding days). We obtained county-level daily maximum temperature data measured as a time-varying covariate from the United States National Centers for Environmental Information Climate Data Online [31]. We obtained zip-code level median household income from the 2000 United States Census [32].

### 2.3. Primary Outcome Measure

The primary outcomes of this study are 30-day readmission from cardiac-related causes and all-cause mortality within 30-days.

### 2.4. Statistical Analysis

We used Fine and Gray regression, a method analogous to the Cox Proportional Hazards model that allows for the determination of the effect of air pollution on the cumulative incidence function of an event in the presence of a competing risk [33,34]. This method was used to analyze the effect of PM_2.5_ exposure (unit of change equals 10 μm/m^3^) on 30-day cardiac readmission hazard rates. Because patients are often readmitted from another cause or die within one year of index admission [16], we adjusted for the competing risk of readmission from another non-cardiovascular cause or death. This allows us to jointly estimate the risk of readmission and mortality. We present results for cardiac-related readmission and all-cause mortality with 3-day average exposure to PM_2.5_, with patients censored at 30 days or time of readmission from another cause, clustered by individual. To investigate sex and age specific PM_2.5_ effects, we stratified analyses by sex (male, female) and age at diagnosis group (65–74, 75–84, 85+). We report hazard ratios and *p*-values for readmission or death. All models controlled for county level maximum daily temperature, frailty as measured by the Charlson Comorbidity Index [35], dual enrollment in Medicare Part A and B, and zip-code level median household income. Results for lag 0, lag 1, and 7-day average exposure measures and Bonferroni corrected estimates (alpha = 0.0125) are found in Supplementary Tables S2–S7. Cause-specific results are shown in Supplementary Tables S8–S13. Analyses were completed using SAS, version 9.4 (SAS Institute, Inc., Cary, NC, USA). This study was approved by the University of Utah Institutional Review Board (IRB_00043524).

## 3. Results

**Figure 1:**
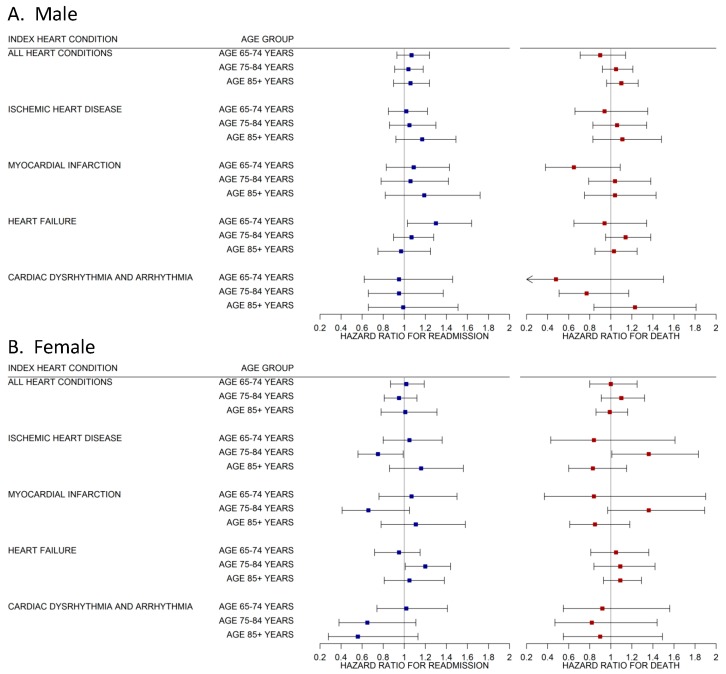
The effect of fine particulate matter (PM_2.5_) air pollution averaged over 3 days on 30-day cardiac readmission and death within 30-days by index admission for cardiovascular patients in Utah’s Medicare population by age group (65–74, 75–84, 85+ years) and sex (male, Panel A; female, Panel B) 1999–2009. Results of Fine and Gray regression. All results jointly estimate the risk of readmission or mortality while adjusting for the competing risk of readmission from a non-cardiac related cause. Results show Bonferroni corrected 98.75% CI’s (alpha = 0.0125). All models adjust for zip code level median-household income, Charlson Comorbidity Index, dual enrollment status, and daily temperature.

Our analysis included 30,510 admission events for 19,602 individuals. Descriptive statistics of the study sample are displayed in Table 1. The sample consisted of almost equal numbers of male and female patients (50.51% and 49.49%, respectively). The majority of the patients were age 75–84 years (45.74%), followed by 65–74 years (29.98%), and 85 years and older (24.28%). Index cardiac admissions included 11,964 admissions for IHD, 4077 for MI, 8378 admissions for HF, and 6146 for cardiac arrhythmias and dysrhythmias. The average follow-up time was 28.07 days (range one to 30 days). Approximately 80.12% of patients were censored at 30 days. There were 2032 cardiac readmissions, 2587 readmissions attributable to non-cardiac causes, and 1420 deaths after discharge. Average PM_2.5_ measures were 10.96 μm/m^3^ 3-day average (standard deviation 9.60 μm/m^3^) and ranged from 0.05 μm/m^3^ to 97.68 μm/m^3^. All results jointly model the risk of readmission while allowing for the competing risk of mortality during the 30 day period. Bonferroni corrected results are displayed in Figure 1.

**Figure 2:**
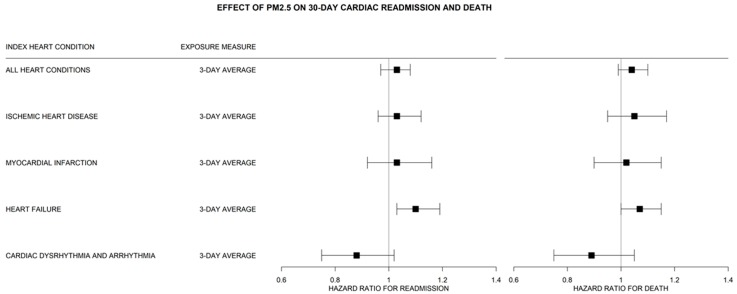
The effect of fine particulate matter (PM_2.5_) air pollution averaged over 3 days on 30-day cardiac readmission and death within 30-days by index admission for cardiovascular patients in Utah’s Medicare population 1999–2009. Results of Fine and Gray regression. All results jointly estimate the risk of readmission or mortality while adjusting for the competing risk of readmission from a non-cardiac related cause. Results show Bonferroni corrected 98.75% CI’s (alpha = 0.0125). All models adjust for zip code level median-household income, Charlson Comorbidity Index, dual enrollment status, and daily temperature.

Results for the cohort are shown in Figure 2 and Table S14. For members of the full cohort admitted for HF, a 10 μm/m^3^ increase in the average exposure to PM_2.5_ across 3 days was associated with a 10% increase in risk of 30-day readmission (*p* = 0.01), however we focus our analysis on sex-and-age-specific effects.

The hazard ratio of readmission from cardiac-related diagnosis or death within 30 days of hospital discharge for male patients are shown in Figure 1, Panel A. Overall for males, we found little association between PM_2.5_ exposure and 30-day readmission or death. Among males age 65–74 years admitted for HF, a 10 μm/m^3^ increase in the average exposure to PM_2.5_ across 3 days was associated with a 30% increase in risk of 30-day readmission (*p* = 0.01). We found no other statistically significant results for males of any age group with index admission attributable to other cardiac-related causes.

Results for female patients are shown in Figure 1, Panel B. We found a stronger association between PM_2.5_ exposure and cardiac related readmission and death in this group. Among females age 75–84 admitted for IHD, a 10 μm/m^3^ increase in 3-day average exposure to PM_2.5_ was associated with a 25% decrease in 30-day readmission (*p* = 0.01), but this was coupled with a 36% increase in the competing risk of death within 30-days (*p* = 0.01). This estimate was largely driven by patients admitted for MI; however the MI specific results were not statistically significant after Bonferroni correction (alpha = 0.0125), which is related to the smaller number of cases. Among females age 75–84 admitted for HF, a 10 μm/m^3^ increase in 3-day average exposure to PM_2.5_ was associated with a 22% increase in 30-day readmission (*p* = 0.01). We found no other statistically significant results for females of any age group with index admission attributable to other cardiac-related causes.

### Supplementary Analysis

We investigated the relationship between PM_2.5_ and other cause readmission adjusted for the competing risk of cardiac readmission and death. We found no statistical association between PM_2.5_ and readmission from a non-cardiac related cause. We investigated the relationship between PM_2.5_ and cardiac readmission without the competing risk of death. A 10 μm/m^3^ increase in 3-day average exposure to PM_2.5_ was associated with a 27% increase in 30-day readmission among males age 65–74 hospitalized for HF. A 10 μm/m^3^ increase in 3-day average exposure to PM_2.5_ was associated with a 25% increase in 30-day readmission among females age 75–84 hospitalized for HF.

## 4. Discussion

Our results show that the relationship between PM_2.5_ exposure and 30-day readmission and mortality is complex and in our analysis varied by index condition, age, and sex. The strongest associations were found in females age 75–84 years, with decreased risk of readmission for those admitted with IHD, but increased risk of death. The mixed evidence in this study underlines the importance of treating the 65 and over population as a heterogeneous group and the necessity of developing studies and possibly interventions that target at risk populations. It also emphasizes the need for air pollution studies that focus on susceptible populations because the current EPA standards may not be low enough to protect those at the highest risk for morbidity and mortality. Methodologically, our study highlights the need to consider the competing risk of death when studying the relationship between air pollution and hospital readmission.

Our findings among patients with IHD and HF are of particular note and may be related to the acuity of the admission. Among males HF of the youngest age group (65–74 years) we found that exposure to PM_2.5_ increased their risk of readmission within 30 days and for men age 75–84, a group with higher baseline mortality than the younger age group, we found that increases in PM_2.5_ exposure were associated with increased mortality, though these results did not reach statistical significance. There was also an association between PM_2.5_ exposure and adverse outcomes following an index MI admission for females. Females age 75–84 were found to have a decreased risk of readmission within 30 days after acute MI, but an increased risk of death. This suggests that PM_2.5_ exposure for females in this age group leads to a more severe outcome in MI patients, possibly due to the acuity of the illness in this group. For conditions with lower mortality, such as HF, high levels of PM_2.5_ were associated with readmission and not death. These findings suggest that individuals with chronic conditions are more likely to be re-admitted when exposed to air pollution, while individuals with an acute cardiac event are more likely to die after exposure, and that this relationship varies by age.

Our findings complement other studies investigating the effect of air pollution on hospital readmissions. Consistent with our findings, von Klot et al. found increased cardiac readmissions among adult MI survivors age 35 and older were associated with same-day concentrations of total particles, PM_10_, CO, NO_2_, and ozone [24]. Our study had key differences in exposure assignment and age classification. von Klot et al. were only able to assign exposure at the city-level and fine particle exposure was estimated retrospectively through particle number concentration, and our measures of air pollution at the zip code level give a more precise definition of exposure. Additionally, the study did not investigate differences in susceptibility by age or sex, and included participants as young as 35. As we have shown, the effect varies by age and understanding the risk by age is important. Liu et al. found also a positive association between PM_2.5_ concentration and readmission among MI survivors [23], which strengthens the support for a true association. Similar to our findings, Pope et al. found an increased risk of readmission for HF patients in the Wasatch Front, however they measured exposure as a 14-day moving average and we investigated acute exposures occurring within 7 days or less [5]. Malik et al. found exposure to PM_2.5_ was associated with worse health status and increased risk of death following MI [26]. These studies in conjunction with our findings support accumulating evidence that air pollution could increase hospital readmissions and practitioners may wish to incorporate these findings into clinical care.

Our study has some notable limitations and strengths. Our exposure measure could underestimate the real exposure because temporal averaging does not replicate sharp peaks in exposure. Furthermore, measurement error exists from zip code level exposure estimates. Additionally, we included PM_2.5_ only in our analyses and we are unable to account for the chemical makeup of PM_2.5_. Because air pollution is a dynamic mixture of various pollutants, multi-pollutant models may provide more insight into the relationship between air quality and readmissions and death. The main drawback of a multipollutant model is the high level of multi-collinearity between pollutants, making it important to examine the association with single pollutant models as well. The strengths of this study include our large cohort size, long study period, our ability to elaborate on differences by sex and age, and topography of the study area which results in high variation of air pollution levels in a short time frame. While many other areas of the US do not experience such acute increases in air pollution, this study adds to literature showing the short term impacts of air pollution on human health. Because of the high baseline mortality rates of Medicare patients, considering the competing risk of death for readmission is essential to understanding the effects of air pollution exposure; patients who die are not at risk for readmission and we suggest Fine and Gray regression be used in research examining environmental exposures and readmission rates. Cause-specific models may not capture the complexity of the association between air pollution exposure and multiple interrelated adverse health outcomes.

Reduction in readmissions has become a national priority, as evidenced by the Hospital Readmissions Reduction Program [20], but in order to reduce readmission rates and healthcare system costs, risk factors must be identified and avoided. Older adults are an extremely heterogeneous group and sex and age differences in risk are important considerations [36]. Older adults have unique and complex medical needs and physiology that is different from younger adults. Those over age 65 are less capable of coping with toxicants, such as air pollution, and chronic conditions may be further exacerbated by environmental exposures. With climate change, additional deaths among older adults due to cardiovascular stress is likely [37] and the interplay between climate change and air pollution is expected to increase pollution-related health events [38]. Climate change is expected to worsen air quality by changing air pollution meteorology, precipitation and removal processes, and altering atmospheric chemistry [39]. Health status of older adults may be further impaired by climate change due to low adaptive capacity and longer cumulative exposure to pollutants. As the number of older adults increases, so does the demand for hospital services for this group [40,41]. Individuals with HF are estimated to have a readmission rate of 22.0% [17]; this study suggests that PM_2.5_ is potentially a modifiable factor in readmission rates for individuals with HF. Targeted interventions that educate sensitive patients about the risk of PM_2.5_ exposure should be developed. These interventions could be aimed at, in addition to lowering PM_2.5_ exposure, behavior change among older adults in the form of complying with alerts to stay indoors when PM_2.5_ levels are elevated will also be important to decrease readmission rates.

Future studies should consider comorbidities and cause of death information in analyses as well as sociological factors including hospital transportation access. Identifying risk factors for the underlying relationship between air pollution, such as life time exposure to pollutants, and cardiovascular outcomes in older adults is of great importance [8,9]. Our study found that some older adult cardiovascular patients may be more sensitive to acute increases in PM_2.5_. Because the EPA gives priority to susceptible subpopulations when assigning limits on air pollutants, it is important to identify these sensitive subpopulations.

## Figures and Tables

**Table 1:** Descriptive Statistics of Sample.

No. of Admissions	30,510
No. of Individuals	19,602
Age Group	
65–74 years	9148(29.98%)
75–84 years	13,954(45.74%)
85 years and older	7408(24.28%)
Sex	
Male	15,411(50.51%)
Female	15,099(49.49%)
Index Admission (not mutually exclusive)	
Myocardial Infarction	4077
Heart Failure	8378
Ischemic Heart Disease	11,964
Cardiac Dysrhythmia and Arrhythmia	6146
Dual Enrollment Status	
Yes	1459(4.78%)
No	29,051(95.22%)
Charlson Comorbidity Index Category	
0	8832(28.95%)
1	7197(23.59%)
2+	14,481(47.46%)
Maximum Daily Temperature	
Mean (Std dev) (degrees F)	58.03(18.76)
Range (degrees F)	13.98–98.05
Median Household Income	
Mean (Std dev) (US dollars)	49,572.83(11,363.21)
Range (US dollars)	22,219.00–87,515.00
PM_2.5_ Measure	
Lag 0	
Mean (Std dev) (μg/m^3^)	10.96(10.45)
Range (μg/m^3^)	0.05–97.68
Lag 1	
Mean (Std dev) (μg/m^3^)	10.95(10.43)
Range (μg/m^3^)	0.05–10.43
3-Day Average	
Mean (Std dev) (μg/m^3^)	10.96(9.60)
Range (μg/m^3^)	0.05–97.68
7-Day Average	
Mean (Std dev) (μg/m^3^)	10.79(10.43)
Range (μg/m^3^)	0.39–79.50
Duration	
Mean(Std dev) (days)	28.07(7.62)
Range(days)	1–30
Outcome	
Cardiac Readmission	2032(6.75%)
Other Cause Readmission	2587(8.48%)
Death	1420(4.65%)
No Readmission	24,471(80.12%)

## Supplementary Materials

The following are available online at https://www.mdpi.com/2077-0383/8/12/2114/s1, Table S1: ICD-9 Codes Included. Tables S2–S7: The effect of fine particulate matter (PM2.5) air pollution on 30-day cardiac readmission and death within 30-days by index admission for cardiovascular patients in Utah’s Medicare population by age group (65–74, 75–84, 85+ years) and sex (male, female) 1999–2009. Tables S8–S13: The effect of fine particulate matter (PM2.5) air pollution on 30-day cardiac readmission within 30-days by index admission for cardiovascular patients in Utah’s Medicare population b by age group (65–74, 75–84, 85+ years) and sex (male, female) 1999–2009. Table S14: The effect of fine particulate matter (PM2.5) air pollution on 30-day cardiac readmission and death within 30-days by index admission for cardiovascular patients in Utah’s Medicare population 1999–2009.
