# Supplementary material for: Evaluation of the Sex-and-Age-Specific Effects of PM2.5 on Hospital Readmission in the Presence of the Competing Risk of Mortality in the Medicare Population of Utah 1999–2009
Source: J Clin Med. 2019 Dec 2;8(12):2114. doi: 10.3390/jcm8122114 (PMC6947183; doi:10.3390/jcm8122114)
Supplement: Supplementary file 1 [file jcm-08-02114-s001.pdf]

Supplementary Table 1: ICD-9 Codes Included	
Any Heart Condition	41000, 41001, 41002, 41010, 41011, 41012, 41020, 41021, 41022, 41031, 41032,41040, 41041, 41042, 41050, 41051, 41052, 41060, 41061, 41062, 41070 ,41071, 41072, 41080, 41081, 41082, 4109, 41090, 41091, 41092, 428,4280,4281,42820,42821,42822,42823,42830,42831,42832,42833,42840, 42841, 42842,42843, 4289, 41000, 41001, 41002, 41010, 41011, 41012, 41020, 41021, 41022, 41031, 41032,41040, 41041, 41042, 41050, 41051, 41052, 41060, 41061, 41062, 41070 ,41071, 41072, 41080, 41081, 41082, 4109, 41090, 41091, 41092, 4110,4111, 41181, 41189, 412, 4130,4131,4139,4140,41400,41401,41402,41403,41404,41405,41406,41407,41410,41411,4143,4148,4149, 4270,4271,4272,42731,42732,42741,42742,4275,42760,42761,42769,4278, 42789, 4279, 4110,4111,41181, 41189, 41511, 4150, 4151, 41519, 4260, 42610,42611,42612,42613,4263,4264,42650,42652,42653,42654,4266, 4267,42682,42689,4269, 4440,4441,44421,44422,44481,44489,4449,452,4530,4531,4532,4533, 45340,45341,45342,45351,45377,4538,45381,45382,45385,4539
MI	41000, 41001, 41002, 41010, 41011, 41012, 41020, 41021, 41022, 41031, 41032,41040, 41041, 41042, 41050, 41051, 41052, 41060, 41061, 41062, 41070 ,41071, 41072, 41080, 41081, 41082, 4109, 41090, 41091, 41092
Heart Failure	428,4280,4281,42820,42821,42822,42823,42830,42831,42832,42833,42840, 42841, 42842,42843, 4289
Ischemic Heart Disease	41000, 41001, 41002, 41010, 41011, 41012, 41020, 41021, 41022, 41031, 41032,41040, 41041, 41042, 41050, 41051, 41052, 41060, 41061, 41062, 41070 ,41071, 41072, 41080, 41081, 41082, 4109, 41090, 41091, 41092, 4110,4111, 41181, 41189, 412, 4130,4131,4139,4140,41400,41401,41402,41403,41404,41405,41406,41407,41410,41411,4143,4148,4149
Cardiac Dysrhythmias/Arrhythmias	4270,4271,4272,42731,42732,42741,42742,4275,42760,42761,42769,4278, 42789, 4279

Tables S2-S7: The effect of fine particulate matter (PM2.5) air pollution on 30-day cardiac readmission and death within 30-days by index admission for cardiovascular patients in Utah's Medicare population by age group (65-74, 75-84, 85+ years) and sex (male, female) 1999-2009. Results of Fine and Gray regression. All results jointly estimate the risk of readmission or mortality while adjusting for the competing risk of readmission from a non-cardiac related cause. Results show 95% confidence intervals (CI) (alpha=0.05) and Bonferroni corrected 98.75% CI's (alpha=0.0125). All models adjust for zip code level median-household income, Charlson Comorbidity Index, dual enrollment status, and daily temperature.

Tables S8-S13: The effect of fine particulate matter (PM2.5) air pollution on 30-day cardiac readmission within 30-days by index admission for cardiovascular patients in Utah's Medicare population b by age group (65-74, 75-84, 85+ years) and sex (male, female) 1999-2009. Results of Cox Proportional Hazards Regression. Results show 95% confidence intervals (CI) (alpha=0.05) and Bonferroni corrected 98.75% CI's (alpha=0.0125). All models adjust for zip code level median-household income, Charlson Comorbidity Index, dual enrollment status, and daily temperature.

Table S14: The effect of fine particulate matter (PM2.5) air pollution on 30-day cardiac readmission and death within 30-days by index admission for cardiovascular patients in Utah's Medicare population 1999-2009. Results of Fine and Gray regression. All results jointly estimate the risk of readmission or mortality while adjusting for the competing risk of readmission from a non-cardiac related cause. Results show 95% confidence intervals (CI) (alpha=0.05) and Bonferroni corrected 98.75% CI's (alpha=0.0125). All models adjust for zip code level median-household income, Charlson Comorbidity Index, dual enrollment status, and daily temperature.

Table S2: Males Age 65-74 Years

Index Heart Condition	Hazard Ratio for Readmission	95% CI	98.75% CI	Pr>ChiSq	Hazard Ratio for Death	95% CI	98.75% CI	Pr>ChiSq
Any Heart Condition								
Lag 0	1.05	0.94,1.17	0.92,1.21	0.36	0.99	0.82,1.19	0.78,1.26	0.90
Lag 1	1.10	0.99,1.23	0.97,1.26	0.07	0.96	0.81,1.14	0.77,1.20	0.65
3-day average	1.08	0.96,1.20	0.91,1.18	0.20	0.90	0.74,1.09	0.71,1.14	0.27
7-day average	1.06	0.93,1.21	0.87,1.18	0.37	0.86	0.69,1.07	0.65,1.13	0.17
Ischemic Heart Disease								
Lag 0	1.04	0.92,1.18	0.89,1.22	0.52	1.05	0.76,1.44	0.70,1.57	0.79
Lag 1	1.04	0.90,1.19	0.87,1.23	0.62	1.06	0.85,1.32	0.80,1.40	0.62
3-day average	1.03	0.90,1.19	0.86,1.23	0.67	0.94	0.71,1.25	0.66,1.35	0.68
7-day average	1.02	0.86,1.21	0.82,1.27	0.79	0.90	0.67,1.22	0.62,1.32	0.50
Myocardial Infarction								
Lag 0	0.97	0.75,1.24	0.70,1.33	0.79	0.77	0.54,1.11	0.49,1.22	0.16
Lag 1	1.09	0.89,1.33	0.84,1.41	0.40	0.80	0.56,1.14	0.51,1.26	0.22
3-day average	1.09	0.88,1.35	0.83,1.43	0.45	0.65	0.43,0.97	0.38,1.09	0.04
7-day average	1.11	0.86,1.43	0.80,1.54	0.43	0.77	0.50,1.19	0.45,1.33	0.24
Heart Failure								
Lag 0	1.26	1.09,1.46	1.05,1.52	<0.01	0.98	0.78,1.23	0.74,1.31	0.88
Lag 1	1.38	1.19,1.59	1.15,1.66	<0.001	0.99	0.75,1.31	0.70,1.41	0.94
3-day average	1.30	1.08,1.56	1.03,1.65	0.01	0.93	0.70,1.24	0.65,1.34	0.63
7-day average	1.23	0.98,1.55	0.92,1.65	0.08	0.92	0.65,1.30	0.59,1.42	0.62
Cardiac Dysrhythmia and Arrhythmia								
Lag 0	0.85	0.53,1.37	0.47,1.56	0.51	0.51	0.24,1.08	0.20,1.32	0.08
Lag 1	1.07	0.82,1.40	0.76,1.50	0.63	0.31	0.12,0.77	0.10,0.98	0.01
3-day average	0.95	0.68,1.33	0.62,1.46	0.79	0.48	0.20,1.18	0.16,1.50	0.11
7-day average	1.01	0.71,1.43	0.65,1.57	0.97	0.46	0.16,1.34	0.12,1.80	0.16

Table S3: Males Age 75-84 Years

Index Heart Condition	Hazard Ratio for Readmission	95% CI	98.75% CI	Pr>ChiSq	Hazard Ratio for Death	95% CI	98.75% CI	Pr>ChiSq
Any Heart Condition								
Lag 0	1.04	0.94,1.15	0.92,1.18	0.41	1.10	1.00,1.21	0.97,1.25	0.06
Lag 1	1.04	0.94,1.16	0.91,1.19	0.42	1.07	0.97,1.19	0.94,1.22	0.18
3-day average	1.04	0.94,1.15	0.91,1.25	0.49	1.05	0.95,1.17	0.92,1.21	0.33
7-day average	1.02	0.90,1.14	0.91,1.30	0.80	1.12	1.00,1.25	0.96,1.29	0.06
Ischemic Heart Disease								
Lag 0	1.05	0.90,1.23	0.86,1.28	0.56	1.11	0.95,1.29	0.91,1.35	0.21
Lag 1	1.06	0.92,1.22	0.88,1.27	0.42	1.08	0.90,1.28	0.86,1.35	0.42
3-day average	1.05	0.90,1.22	0.86,1.28	0.54	1.06	0.88,1.27	0.84,1.34	0.56
7-day average	1.05	0.89,1.24	0.86,1.30	0.54	1.20	0.99,1.45	0.93,1.53	0.07
Myocardial Infarction								
Lag 0	1.04	0.81,1.34	0.76,1.44	0.74	1.09	0.90,1.33	0.85,1.41	0.37
Lag 1	1.01	0.80,1.27	0.75,1.35	0.95	1.01	0.82,1.26	0.77,1.33	0.91
3-day average	1.06	0.84,1.33	0.78,1.42	0.65	1.04	0.84,1.30	0.79,1.38	0.70
7-day average	1.06	0.83,1.36	0.77,1.45	0.66	1.22	0.99,1.50	0.93,1.59	0.07
Heart Failure								
Lag 0	1.10	0.96,1.25	0.93,1.29	0.17	1.15	0.99,1.33	0.95,1.39	0.08
Lag 1	1.06	0.91,1.23	0.87,1.28	0.45	1.16	1.01,1.32	0.98,1.37	0.03
3-day average	1.07	0.93,1.23	0.90,1.28	0.31	1.14	0.99,1.32	0.95,1.38	0.08
7-day average	1.04	0.88,1.22	0.85,1.27	0.66	1.16	0.99,1.35	0.95,1.41	0.07
Cardiac Dysrhythmia and Arrhythmia								
Lag 0	0.97	0.74,1.29	0.68,1.39	0.85	0.99	0.78,1.25	0.73,1.33	0.91
Lag 1	0.95	0.72,1.25	0.67,1.35	0.72	0.70	0.46,1.07	0.41,1.20	0.10
3-day average	0.95	0.71,1.26	0.66,1.37	0.72	0.77	0.55,1.07	0.51,1.17	0.12
7-day average	0.90	0.61,1.33	0.55,1.48	0.59	0.87	0.57,1.32	0.51,1.48	0.52

Table S4: Males Age 85+ Years

Index Heart Condition	Hazard Ratio for Readmission	95% CI	98.75% CI	Pr>ChiSq	Hazard Ratio for Death	95% CI	98.75% CI	Pr>ChiSq
Any Heart Condition								
Lag 0	0.98	0.86,1.13	0.82,1.17	0.79	1.05	0.94,1.18	0.91,1.22	0.39
Lag 1	1.06	0.94,1.20	0.91,1.24	0.32	1.10	0.99,1.23	0.96,1.26	0.09
3-day average	1.06	0.94,1.21	0.87,1.19	0.34	1.10	0.99,1.23	0.96,1.26	0.09
7-day average	1.09	0.95,1.25	0.83,1.22	0.24	1.07	0.93,1.22	0.90,1.27	0.36
Ischemic Heart Disease								
Lag 0	1.06	0.87,1.30	0.82,1.38	0.56	1.11	0.91,1.35	0.86,1.42	0.32
Lag 1	1.04	0.85,1.27	0.81,1.34	0.69	1.05	0.84,1.31	0.79,1.39	0.68
3-day average	1.17	0.97,1.42	0.92,1.49	0.11	1.11	0.89,1.40	0.83,1.48	0.35
7-day average	1.22	0.99,1.49	0.94,1.57	0.06	1.08	0.83,1.42	0.77,1.53	0.56
Myocardial Infarction								
Lag 0	1.07	0.78,1.45	0.72,1.58	0.68	1.04	0.82,1.32	0.77,1.41	0.75
Lag 1	0.99	0.70,1.40	0.64,1.54	0.95	0.95	0.73,1.23	0.68,1.33	0.69
3-day average	1.19	0.89,1.59	0.82,1.72	0.25	1.04	0.81,1.33	0.75,1.43	0.77
7-day average	1.18	0.85,1.65	0.77,1.81	0.33	1.05	0.79,1.38	0.73,1.49	0.75
Heart Failure								
Lag 0	0.93	0.76,1.14	0.71,1.21	0.49	0.96	0.80,1.15	0.76,1.21	0.66
Lag 1	1.09	0.91,1.29	0.87,1.35	0.35	1.08	0.94,1.25	0.90,1.30	0.28
3-day average	0.98	0.80,1.20	0.75,1.27	0.83	1.03	0.89,1.20	0.85,1.25	0.67
7-day average	0.95	0.74,1.21	0.69,1.29	0.65	0.99	0.82,1.20	0.78,1.26	0.94
Cardiac Dysrhythmia and Arrhythmia								
Lag 0	0.79	0.50,1.24	0.44,1.41	0.30	1.19	0.88,1.62	0.81,1.76	0.25
Lag 1	1.03	0.77,1.37	0.71,1.48	0.86	1.08	0.80,1.47	0.74,1.59	0.60
3-day average	0.99	0.72,1.38	0.66,1.51	0.97	1.23	0.92,1.66	0.84,1.81	0.17
7-day average	1.09	0.81,1.47	0.74,1.60	0.57	1.24	0.82,1.87	0.73,2.09	0.31

Table S5: Females Age 65-74 Years

Index Heart Condition	Hazard Ratio for Readmission	95% CI	98.75% CI	Pr>ChiSq	Hazard Ratio for Death	95% CI	98.75% CI	Pr>ChiSq
Any Heart Condition								
Lag 0	1.04	0.92,1.18	0.89,1.22	0.54	1.00	0.85,1.18	0.81,1.24	0.96
Lag 1	1.03	0.91,1.15	0.88,1.19	0.67	1.01	0.85,1.20	0.81,1.26	0.92
3-day average	1.02	0.90,1.15	0.81,1.12	0.76	1.00	0.84,1.19	0.80,1.25	0.99
7-day average	1.01	0.86,1.17	0.86,1.19	0.94	0.93	0.74,1.15	0.70,1.22	0.49
Ischemic Heart Disease								
Lag 0	1.01	0.80,1.28	0.75,1.36	0.92	0.67	0.28,1.60	0.22,2.03	0.36
Lag 1	1.03	0.83,1.28	0.78,1.36	0.80	0.70	0.33,1.47	0.27,1.81	0.34
3-day average	1.04	0.84,1.29	0.80,1.37	0.69	0.84	0.50,1.40	0.44,1.61	0.50
7-day average	1.09	0.86,1.37	0.81,1.46	0.47	0.64	0.29,1.39	0.24,1.72	0.26
Myocardial Infarction								
Lag 0	0.92	0.63,1.35	0.57,1.50	0.67	0.24	0.03,1.88	0.02,3.31	0.17
Lag 1	0.78	0.52,1.17	0.46,1.31	0.23	0.39	0.10,1.57	0.07,2.30	0.19
3-day average	1.07	0.82,1.40	0.76,1.5	0.61	0.84	0.44,1.59	0.37,1.90	0.59
7-day average	1.23	0.95,1.61	0.88,1.73	0.12	0.53	0.13,2.13	0.09,3.13	0.37
Heart Failure								
Lag 0	0.91	0.70,1.2	0.65,1.29	0.52	1.04	0.88,1.25	0.83,1.31	0.63
Lag 1	1.01	0.84,1.21	0.79,1.27	0.95	1.05	0.86,1.28	0.81,1.36	0.65
3-day average	0.95	0.77,1.19	0.72,1.26	0.67	1.05	0.85,1.29	0.81,1.36	0.66
7-day average	0.95	0.73,1.23	0.68,1.31	0.68	1.00	0.79,1.28	0.73,1.37	0.97
Cardiac Dysrhythmia and Arrhythmia								
Lag 0	1.17	0.96,1.43	0.91,1.51	0.11	0.97	0.60,1.59	0.52,1.82	0.92
Lag 1	1.04	0.84,1.30	0.79,1.38	0.71	0.90	0.59,1.39	0.52,1.57	0.64
3-day average	1.03	0.80,1.33	0.74,1.42	0.83	0.92	0.61,1.39	0.55,1.56	0.70
7-day average	0.92	0.67,1.25	0.62,1.36	0.58	0.75	0.43,1.33	0.36,1.56	0.33

Table S6: Females Age 75-84 Years

Index Heart Condition	Hazard Ratio for Readmission	95% CI	98.75% CI	Pr>ChiSq	Hazard Ratio for Death	95% CI	98.75% CI	Pr>ChiSq
Any Heart Condition								
Lag 0	0.92	0.80,1.05	0.77,1.09	0.21	1.12	0.99,1.28	0.95,1.33	0.08
Lag 1	1.02	0.91,1.15	0.83,1.30	0.70	1.08	0.95,1.24	0.91,1.29	0.25
3-day average	0.95	0.84,1.08	0.77,1.31	0.47	1.10	0.95,1.27	0.91,1.32	0.21
7-day average	1.01	0.89,1.15	0.69,1.31	0.86	1.10	0.94,1.30	0.89,1.36	0.24
Ischemic Heart Disease								
Lag 0	0.78	0.61,1.01	0.57,1.08	0.05	1.36	1.12,1.65	1.07,1.74	<0.01
Lag 1	0.75	0.59,0.96	0.55,1.02	0.02	1.35	1.05,1.73	0.98,1.86	0.02
3-day average	0.75	0.60,0.94	0.56,1.00	0.01	1.36	1.08,1.71	1.01,1.83	0.01
7-day average	0.88	0.69,1.12	0.65,1.19	0.30	1.35	1.03,1.78	0.95,1.92	0.03
Myocardial Infarction								
Lag 0	0.71	0.49,1.04	0.44,1.15	0.08	1.35	1.06,1.74	0.99,1.86	0.02
Lag 1	0.69	0.47,1.01	0.42,1.12	0.05	1.35	1.02,1.78	0.95,1.93	0.03
3-day average	0.66	0.46,0.95	0.41,1.05	0.03	0.84	0.44,1.59	0.97,1.89	0.02
7-day average	0.64	0.44,0.92	0.40,1.02	0.02	1.31	0.94,1.81	0.86,1.98	0.11
Heart Failure								
Lag 0	1.14	0.97,1.34	0.92,1.40	0.12	1.08	0.87,1.33	0.83,1.41	0.47
Lag 1	1.32	1.15,1.52	1.11,1.58	<0.001	1.07	0.89,1.29	0.85,1.36	0.45
3-day average	1.22	1.06,1.40	1.02,1.46	0.01	1.09	0.88,1.34	0.84,1.42	0.42
7-day average	1.24	1.06,1.46	1.01,1.53	0.01	1.10	0.87,1.38	0.82,1.48	0.43
Cardiac Dysrhythmia and Arrhythmia								
Lag 0	0.61	0.42,0.91	0.38,1.01	0.01	1.08	0.78,1.49	0.72,1.62	0.65
Lag 1	0.77	0.52,1.12	0.47,1.24	0.17	0.79	0.54,1.16	0.49,1.29	0.23
3-day average	0.65	0.42,0.99	0.38,1.11	0.04	0.82	0.53,1.27	0.47,1.44	0.38
7-day average	0.73	0.50,1.07	0.45,1.18	0.10	0.97	0.58,1.62	0.51,1.87	0.92

Table S7: Females Age 85+ Years

Index Heart Condition	Hazard Ratio for Readmission	95% CI	98.75% CI	Pr>ChiSq	Hazard Ratio for Death	95% CI	98.75% CI	Pr>ChiSq
Any Heart Condition								
Lag 0	1.04	0.87,1.24	0.83,1.30	0.66	1.00	0.89,1.12	0.86,1.15	0.98
Lag 1	1.04	0.87,1.24	0.93,1.24	0.69	1.06	0.95,1.17	0.92,1.21	0.31
3-day average	1.01	0.82,1.23	0.90,1.26	0.96	1.00	0.89,1.12	0.86,1.16	1.00
7-day average	0.95	0.73,1.22	0.70,1.33	0.67	1.00	0.87,1.13	0.84,1.17	0.94
Ischemic Heart Disease								
Lag 0	1.16	0.95,1.43	0.90,1.51	0.15	0.83	0.63,1.09	0.58,1.17	0.17
Lag 1	1.18	0.97,1.44	0.92,1.52	0.10	0.89	0.72,1.10	0.68,1.16	0.27
3-day average	1.15	0.90,1.47	0.84,1.57	0.27	0.84	0.65,1.08	0.61,1.15	0.16
7-day average	1.13	0.85,1.49	0.79,1.61	0.40	0.92	0.71,1.21	0.66,1.30	0.56
Myocardial Infarction								
Lag 0	1.08	0.83,1.41	0.77,1.52	0.56	0.83	0.63,1.09	0.58,1.18	0.18
Lag 1	1.10	0.84,1.44	0.78,1.55	0.50	0.87	0.69,1.10	0.65,1.17	0.25
3-day average	1.07	0.79,1.43	0.73,1.55	0.67	0.85	0.66,1.10	0.61,1.19	0.23
7-day average	1.03	0.73,1.44	0.67,1.58	0.88	0.94	0.71,1.24	0.65,1.34	0.65
Heart Failure								
Lag 0	1.07	0.89,1.30	0.84,1.37	0.47	1.09	0.96,1.23	0.93,1.27	0.17
Lag 1	1.07	0.87,1.32	0.82,1.40	0.53	1.15	1.02,1.29	0.99,1.33	0.02
3-day average	1.05	0.85,1.30	0.80,1.38	0.64	1.09	0.96,1.24	0.93,1.29	0.18
7-day average	0.96	0.72,1.28	0.66,1.39	0.78	1.06	0.91,1.24	0.88,1.29	0.42
Cardiac Dysrhythmia and Arrhythmia								
Lag 0	0.58	0.29,1.16	0.24,1.40	0.12	0.91	0.63,1.31	0.57,1.45	0.62
Lag 1	0.68	0.46,1.01	0.41,1.13	0.06	0.73	0.40,1.32	0.34,1.55	0.30
3-day average	0.57	0.33,0.99	0.28,1.16	0.05	0.90	0.61,1.34	0.55,1.49	0.62
7-day average	0.46	0.22,0.93	0.18,1.14	0.03	0.89	0.58,1.35	0.52,1.51	0.58

Table S8: Cause Specific Models: Males Age 65-74 Years

Index Heart Condition	Hazard Ratio for Readmission	95% CI	98.75% CI	Pr>ChiSq
Any Heart Condition				
Lag 0	1.06	0.95,1.18	0.93,1.21	0.28
Lag 1	1.11	1.00,1.22	0.97,1.26	0.06
3-day average	1.08	0.97,1.20	0.94,1.24	0.16
7-day average	1.08	0.94,1.22	0.91,1.26	0.28
Ischemic Heart Disease				
Lag 0	1.06	0.94,1.20	0.91,1.24	0.35
Lag 1	1.05	0.92,1.20	0.89,1.25	0.47
3-day average	1.04	0.91,1.20	0.88,1.24	0.54
7-day average	1.04	0.88,1.23	0.85,1.28	0.62
Myocardial Infarction				
Lag 0	1.00	0.81,1.24	0.77,1.31	0.97
Lag 1	1.12	0.94,1.33	0.89,1.40	0.22
3-day average	1.11	0.92,1.34	0.87,1.42	0.29
7-day average	1.14	0.90,1.44	0.84,1.53	0.29
Heart Failure				
Lag 0	1.23	1.07,1.42	1.03,1.47	<0.01
Lag 1	1.34	1.16,1.55	1.12,1.61	<0.001
3-day average	1.27	1.07,1.50	1.02,1.57	<0.01
7-day average	1.20	0.97,1.49	0.92,1.58	0.09
Cardiac Dysrhythmia and Arrhythmia				
Lag 0	0.87	0.56,1.35	0.50,1.53	0.54
Lag 1	1.05	0.81,1.37	0.75,1.47	0.71
3-day average	0.96	0.70,1.32	0.64,1.45	0.81
7-day average	1.02	0.73,1.41	0.67,1.54	0.93

Table S9: Cause Specific Models: Males Age 75-84 Years

Index Heart Condition	Hazard Ratio for Readmission	95% CI	98.75% CI	Pr>ChiSq
Any Heart Condition				
Lag 0	1.05	0.95,1.15	0.92,1.19	0.36
Lag 1	1.05	0.95,1.16	0.92,1.19	0.39
3-day average	1.04	0.94,1.15	0.92,1.19	0.41
7-day average	1.03	0.92,1.15	0.89,1.19	0.64
Ischemic Heart Disease				
Lag 0	1.05	0.91,1.22	0.87,1.28	0.50
Lag 1	1.05	0.92,1.22	0.88,1.26	0.46
3-day average	1.05	0.90,1.22	0.87,1.27	0.53
7-day average	1.05	0.90,1.24	0.86,1.29	0.52
Myocardial Infarction				
Lag 0	1.07	0.83,1.38	0.77,1.48	0.61
Lag 1	1.01	0.80,1.27	0.76,1.35	0.92
3-day average	1.06	0.84,1.34	0.79,1.42	0.62
7-day average	1.06	0.85,1.33	0.80,1.42	0.60
Heart Failure				
Lag 0	1.10	0.96,1.25	0.93,1.30	0.16
Lag 1	1.06	0.92,1.24	0.88,1.29	0.42
3-day average	1.08	0.94,1.25	0.91,1.29	0.27
7-day average	1.05	0.89,1.23	0.85,1.29	0.56
Cardiac Dysrhythmia and Arrhythmia				
Lag 0	0.98	0.74,1.30	0.69,1.41	0.91
Lag 1	0.95	0.71,1.25	0.66,1.35	0.70
3-day average	0.95	0.71,1.28	0.66,1.38	0.75
7-day average	0.92	0.62,1.36	0.55,1.52	0.67

Table S10: Cause Specific Models :Males Age 75-84 Years

Index Heart Condition	Hazard Ratio for Readmission	95% CI	98.75% CI	Pr>ChiSq
Any Heart Condition				
Lag 0	0.97	0.85,1.12	0.81,1.16	0.69
Lag 1	1.05	0.93,1.20	0.90,1.24	0.43
3-day average	1.06	0.93,1.21	0.90,1.26	0.36
7-day average	1.08	0.93,1.25	0.89,1.30	0.32
Ischemic Heart Disease				
Lag 0	1.07	0.88,1.31	0.83,1.39	0.49
Lag 1	1.05	0.86,1.28	0.82,1.36	0.62
3-day average	1.17	0.97,1.42	0.93,1.49	0.09
7-day average	1.22	1.00,1.48	0.95,1.57	0.05
Myocardial Infarction				
Lag 0	1.08	0.80,1.45	0.74,1.57	0.63
Lag 1	0.99	0.70,1.40	0.64,1.53	0.96
3-day average	1.19	0.90,1.58	0.83,1.71	0.22
7-day average	1.19	0.86,1.64	0.79,1.79	0.30
Heart Failure				
Lag 0	0.88	0.71,1.09	0.67,1.16	0.25
Lag 1	1.06	0.88,1.29	0.83,1.36	0.54
3-day average	0.94	0.76,1.17	0.72,1.24	0.59
7-day average	0.88	0.67,1.15	0.63,1.23	0.34
Cardiac Dysrhythmia and Arrhythmia				
Lag 0	0.79	0.51,1.22	0.45,1.38	0.28
Lag 1	1.01	0.76,1.35	0.70,1.46	0.93
3-day average	0.99	0.71,1.36	0.65,1.49	0.93
7-day average	1.08	0.80,1.46	0.73,1.58	0.63

Table S11: Cause Specific Models: Females Age 65-74 Years

Index Heart Condition	Hazard Ratio for Readmission	95% CI	98.75% CI	Pr>ChiSq
Any Heart Condition				
Lag 0	1.04	0.92,1.18	0.89,1.22	0.50
Lag 1	1.03	0.92,1.16	0.89,1.20	0.59
3-day average	1.02	0.91,1.16	0.88,1.20	0.70
7-day average	1.01	0.87,1.17	0.83,1.22	0.91
Ischemic Heart Disease				
Lag 0	1.03	0.82,1.28	0.78,1.36	0.80
Lag 1	1.02	0.82,1.27	0.77,1.35	0.85
3-day average	1.05	0.85,1.29	0.80,1.36	0.67
7-day average	1.08	0.86,1.36	0.80,1.45	0.53
Myocardial Infarction				
Lag 0	0.92	0.62,1.36	0.55,1.52	0.67
Lag 1	0.75	0.49,1.16	0.43,1.30	0.19
3-day average	1.04	0.78,1.38	0.72,1.49	0.81
7-day average	1.16	0.88,1.54	0.82,1.66	0.29
Heart Failure				
Lag 0	0.92	0.70,1.20	0.65,1.29	0.56
Lag 1	1.02	0.85,1.22	0.81,1.28	0.86
3-day average	0.96	0.78,1.19	0.73,1.26	0.72
7-day average	0.95	0.74,1.23	0.69,1.32	0.71
Cardiac Dysrhythmia and Arrhythmia				
Lag 0	1.17	0.96,1.41	0.91,1.49	0.13
Lag 1	1.04	0.84,1.29	0.79,1.37	0.71
3-day average	1.03	0.80,1.32	0.75,1.41	0.84
7-day average	0.91	0.67,1.24	0.62,1.35	0.56

Table S12: Cause Specific Models: Females Age 75-84 Years

Index Heart Condition	Hazard Ratio for Readmission	95% CI	98.75% CI	Pr>ChiSq
Any Heart Condition				
Lag 0	0.91	0.79,1.05	0.76,1.09	0.19
Lag 1	1.02	0.90,1.16	0.87,1.20	0.72
3-day average	0.95	0.83,1.08	0.80,1.12	0.44
7-day average	1.01	0.88,1.15	0.85,1.20	0.89
Ischemic Heart Disease				
Lag 0	0.73	0.55,0.97	0.51,1.05	0.03
Lag 1	0.73	0.56,0.95	0.52,1.02	0.02
3-day average	0.71	0.56,0.92	0.52,0.98	0.01
7-day average	0.86	0.66,1.12	0.61,1.20	0.26
Myocardial Infarction				
Lag 0	1.07	0.64,0.41	0.36,1.15	0.06
Lag 1	1.01	0.64,0.41	0.37,1.14	0.05
3-day average	1.06	0.62,0.41	0.36,1.06	0.03
7-day average	1.06	0.59,0.39	0.35,1.02	0.02
Heart Failure				
Lag 0	1.15	0.98,1.35	0.93,1.41	0.09
Lag 1	1.33	1.16,1.53	1.12,1.58	<0.001
3-day average	1.25	1.08,1.44	1.04,1.50	<0.01
7-day average	1.28	1.08,1.50	1.04,1.57	<0.01
Cardiac Dysrhythmia and Arrhythmia				
Lag 0	0.63	0.43,0.92	0.38,1.03	0.02
Lag 1	0.78	0.54,1.13	0.49,1.25	0.19
3-day average	0.66	0.43,1.00	0.39,1.12	0.05
7-day average	0.74	0.51,1.08	0.46,1.19	0.17

Table S13: Cause Specific Models: Females Age 85+ Years

Index Heart Condition	Hazard Ratio for Readmission	95% CI	98.75% CI	Pr>ChiSq
Any Heart Condition				
Lag 0	1.03	0.89,1.19	0.86,1.24	0.69
Lag 1	1.03	0.89,1.20	0.86,1.24	0.66
3-day average	0.99	0.83,1.19	0.79,1.25	0.94
7-day average	0.93	0.74,1.17	0.69,1.24	0.53
Ischemic Heart Disease				
Lag 0	1.11	0.96,1.29	0.92,1.35	0.17
Lag 1	1.14	0.98,1.32	0.94,1.37	0.08
3-day average	1.10	0.92,1.30	0.88,1.37	0.31
7-day average	1.09	0.88,1.36	0.82,1.44	0.44
Myocardial Infarction				
Lag 0	1.07	0.82,1.38	0.77,1.48	0.63
Lag 1	1.09	0.84,1.42	0.78,1.52	0.50
3-day average	1.05	0.79,1.41	0.73,1.53	0.72
7-day average	1.02	0.72,1.44	0.66,1.59	0.91
Heart Failure				
Lag 0	1.07	0.88,1.30	0.83,1.37	0.49
Lag 1	1.06	0.85,1.32	0.80,1.40	0.60
3-day average	1.03	0.83,1.28	0.78,1.36	0.79
7-day average	0.92	0.68,1.25	0.62,1.36	0.60
Cardiac Dysrhythmia and Arrhythmia				
Lag 0	0.60	0.30,1.20	0.25,1.46	0.15
Lag 1	0.70	0.48,1.03	0.43,1.15	0.07
3-day average	0.58	0.33,1.02	0.28,1.19	0.06
7-day average	0.45	0.21,0.96	0.17,1.18	0.04

Table S14: Cohort

Index Heart Condition	Hazard Ratio for Readmission	95% CI	98.75% CI	Pr>ChiSq	Hazard Ratio for Death	95% CI	98.75% CI	Pr>ChiSq
Any Heart Condition								
Lag 0	1.02	0.96,1.07	0.95,1.09	0.54	1.05	1.00,1.11	0.99,1.13	0.05
Lag 1	1.05	1.00,1.11	0.99,1.12	0.05	1.06	1.01,1.12	0.99,1.13	0.02
3-day average	1.03	0.97,1.08	0.96,1.10	0.33	1.04	0.99,1.10	0.97,1.12	0.14
7-day average	1.02	0.96,1.09	0.95,1.10	0.47	1.04	0.98,1.11	0.96,1.13	0.22
Ischemic Heart Disease								
Lag 0	1.03	0.96,1.12	0.94,1.14	0.40	1.08	0.98,1.19	0.96,1.23	0.11
Lag 1	1.03	0.95,1.11	0.93,1.13	0.49	1.07	0.97,1.18	0.94,1.22	0.18
3-day average	1.03	0.96,1.12	0.93,1.14	0.42	1.05	0.95,1.17	0.92,1.20	0.32
7-day average	1.05	0.97,1.15	0.94,1.17	0.24	1.10	0.98,1.23	0.95,1.27	0.10
Myocardial Infarction								
Lag 0	0.99	0.87,1.11	0.85,1.15	0.81	1.02	0.91,1.15	0.88,1.19	0.73
Lag 1	0.98	0.88,1.10	0.85,1.14	0.76	1.00	0.88,1.13	0.85,1.17	0.96
3-day average	1.03	0.92,1.16	0.90,1.19	0.56	1.02	0.90,1.15	0.87,1.19	0.80
7-day average	1.04	0.92,1.18	0.89,1.22	0.52	1.08	0.95,1.23	0.92,1.28	0.23
Heart Failure								
Lag 0	1.08	1.01,1.16	0.99,1.19	0.03	1.06	0.99,1.13	0.97,1.16	0.11
Lag 1	1.16	1.08,1.24	1.06,1.26	<0.0001	1.10	1.03,1.17	1.01,1.19	<0.01
3-day average	1.10	1.03,1.19	1.01,1.21	0.01	1.07	1.00,1.15	0.98,1.17	0.06
7-day average	1.07	0.99,1.17	0.96,1.20	0.1	1.05	0.97,1.14	0.95,1.17	0.21
Cardiac Dysrhythmia and Arrhythmia								
Lag 0	0.86	0.74,1.01	0.71,1.06	0.07	0.99	0.85,1.15	0.82,1.20	0.92
Lag 1	0.94	0.82,1.07	0.79,1.11	0.32	0.79	0.65,0.96	0.62,1.02	0.02
3-day average	0.88	0.75,1.02	0.72,1.06	0.08	0.89	0.75,1.05	0.71,1.10	0.17
7-day average	0.87	0.74,1.03	0.70,1.08	0.11	0.91	0.74,1.12	0.69,1.18	0.36
